# Functional characterization of spike RBD mutations in SARS-CoV-2 Omicron-derived subvariants KP.3.1.1, LP.8.1, and NB.1.8.1.

**DOI:** 10.71150/jm.2511014

**Published:** 2026-04-06

**Authors:** Yeong Jun Kim, Seon Jae Jeong, Hye-Ra Lee

**Affiliations:** 1 Department of Biotechnology and Bioinformatics, College of Science and Technology, Korea University, Sejong 30019, Republic of Korea; 2 Department of Lab Medicine, College of Medicine, Korea University, Seoul 08308, Republic of Korea

**Keywords:** SARS-CoV-2, Omicron, KP.3.1.1, LP.8.1, NB.1.8.1, spike RBD mutation, pseudovirus

## Abstract

Following the global spread of SARS-CoV-2 Omicron (B.1.1.529), its subvariants KP.3.1.1, LP.8.1, and NB.1.8.1 disseminated worldwide. By April 2025, the epidemiological landscape of these subvariants had become distinct, with LP.8.1 emerging as the predominant variant, KP.3.1.1 persisting as a co-circulating variant under monitoring (VUM), and NB.1.8.1 exhibiting a significant increase in prevalence. Despite their epidemiological prominence, the functional consequences of spike mutations defining these emerging subvariants remain poorly understood. Here, we systematically dissected the entry properties conferred by their receptor-binding domain (RBD) mutations using a pseudovirus system. Our results demonstrate that all three subvariants exhibited substantially higher infectivity than ancestral Omicron. Unexpectedly, this enhanced infectivity occurred despite reduced ACE2 binding affinity. Rather, increased viral entry consistently correlated with elevated spike cleavage efficiency and fusogenicity, suggesting a compensatory evolutionary strategy in which enhanced spike processing and fusion contribute to enhanced entry despite reduced receptor engagement. These findings provide a virological explanation for the accelerated global spread of these subvariants and highlight the importance of monitoring functional shifts in spike-mediated entry that may influence SARS-CoV-2 transmission dynamics.

## Introduction

The continual evolution of SARS-CoV-2, characterized by waves of novel emerging variants, is primarily driven by functional adaptations within its spike glycoprotein (Tao et al., 2021). As a trimeric class I fusion protein, spike orchestrates viral entry through a multi-step activation cascade (Walls et al., 2020). This entry process is initiated when the S1 subunit’s receptor-binding domain (RBD) engages the host receptor ACE2 (Zhou et al., 2020). Subsequently, the spike is proteolytically primed at the S1/S2 polybasic site by host furin. This initial cleavage is a prerequisite for a second cleavage event at the S2' site, which exposes the fusion peptide within the S2 subunit and triggers membrane fusion of viral and cellular membranes (Hoffmann et al., 2020). As the primary interface for both receptor binding and antibody neutralization, the spike’s RBD is a focal point of intense and conflicting selective pressures. This evolutionary dynamic has been most pronounced in the Omicron lineage, which emerged in late 2021 and rapidly diversified into numerous sublineages with distinct virologic features (Willett et al., 2022).

Early Omicron sublineages, such as BA.1 and BA.2, were followed in August 2023 by BA.2.86, a highly divergent BA.2 descendant. This variant bore over 30 substitutions concentrated in the spike protein, indicating potential shifts in infectivity and antigenicity. The rapid diversification of BA.2.86 led to the emergence of further sublineages through 2024 and 2025. Among these, KP.3.1.1 was designated a variant under monitoring (VUM) in July 2024 due to its growing transmission. Subsequently, another descendant lineage LP.8.1 disseminated widely and emerged as the predominant variant by April 2025, accounting for approximately 30% of global detections. Concurrently, NB.1.8.1 also increased in prevalence across multiple regions, prompting its own designation as a VUM on May 2025. However, the functional impact of these recent genetic changes on receptor binding, membrane fusion, and infectivity has not been comprehensively defined.

In this study, we functionally characterized the RBD mutations identified in these emerging Omicron subvariants. We first observed that pseudoviruses bearing the spike proteins of KP.3.1.1, LP.8.1, and NB.1.8.1 exhibited markedly higher infectivity than ancestral Omicron. Given that enhanced infectivity is typically driven by increased receptor engagement, we next evaluated their ACE2 binding affinity. Unexpectedly, all three subvariant spikes displayed reduced affinity relative to ancestral Omicron. This discrepancy prompted us to explore post-binding events that might account for the enhanced entry phenotype. We found that enhanced infectivity was consistently associated with elevated spike cleavage efficiency and augmented fusogenicity. Together, these findings provide a virological basis for the epidemiological success of these subvariants, elucidating a compensatory evolutionary strategy that supports their rapid global dissemination.

## Materials and Methods

### Cell lines

293T (ATCC; CRL-3216), 293T-ACE2-TMPRSS2, and Caco-2 (ATCC; HTB-37) cells were cultured in Dulbecco’s Modified Eagle’s Medium (DMEM) with 10% fetal bovine serum (FBS) and 1% penicillin-streptomycin (P/S) (Hyclone) at 37°C under 5% CO_2_. A 293T cell line stably expressing ACE2 and TMPRSS2 was established by transfecting 293T cells with pCEP4-myc-hACE2 (Addgene; 141185) and pCDH-3xFlag-TMPRSS2 using PEI (Sigma) and selection with Hygromycin B and Puromycin (Invitrogen).

### Plasmid construction

The plasmid encoding SARS-CoV-2 Spike harboring RBD mutations of Omicron subvariants (KP.3.1.1, LP.8.1, and NB.1.8.1) were generated using SARS-CoV-2 Omicron RBD mutant Spike as a template by a Quikchange XL site-directed mutagenesis kit (Agilent; 200521). The primers used for site-directed mutagenesis of SARS-CoV-2 Spike RBD mutants are listed on Table 1.

### Chemicals and antibodies

Materials used in this study were purchased from the following manufacturers: Bright-Glo and FuGENE HD from Promega; Polyethylenimine (PEI) from Sigma; PEIpro from Polyplus; RNAiso Plus from TaKaRa; TPCK-trypsin from Thermo Fisher; SsoAdvanced Universal SYBR Green Supermix from Bio-Rad. Recombinant Human ACE-2 His-tag Biotinylated Protein from R&D Systems; Streptavidin APC conjugate from Invitrogen; Anti-SARS-CoV-2 Spike (GTX632604) from GeneTex, and the anti-β-actin antibody (A5316) from Sigma.

### Pseudovirus production and titration

Retrovirus-based pseudotyped viruses harboring SARS-CoV-2 spike D614G or RBD mutations from Omicron and its subvariants were generated as described previously (Kim et al., 2021). Briefly, 293T cells transfected with individual SARS-CoV-2 RBD mutant spike of Omicron subvariants, MLV gag/pol, and pQCXIP-fLuc encoding plasmids using PEI pro (Millet and Whittaker, 2016). After 48 h of transfection, culture supernatants containing the pseudoviruses were harvested and stored at 4°C. Titration of pseudoviruses was measured by quantitative RT-PCR. 300 μl of the individual pseudoviral supernatants were used for extraction of total RNA sample using RNAiso Plus (TaKaRa) according to the manufacturer’s instruction. cDNA was synthesized from each RNA sample using a cDNA synthesis kit (Toyobo), and quantitative RT-PCR was conducted using SsoAdvanced Universal SYBR Green Supermix on the CFX96 Real-Time System (Bio-Rad). To detect the pQCXIP-fLuc reporter plasmid from pseudoviral supernatant, a primer pair targeting the MLV 3′ LTR region was used: Forward 5′-ATTGACTGAGTCGCCCGG-3′; Reverse 5′-AGCGAGACCACAAGTCGGAT-3′. The copy number of pQCXIP-fLuc in each pseudovirus sample was calculated based on a standard curve generated from serially diluted pQCXIP-fLuc plasmid.

### Measurement of infectivity

Using quantitative RT-PCR, the pseudoviruses were normalized to equivalent amounts based on RNA copy numbers of individual samples. After normalization, pseudoviruses harboring spike proteins of individual RBD mutations were transduced into 293T-ACE2 and Caco-2 cells. Subsequently, 24 h after transduction, Bright-Glo was added to the cells and relative luminescence units (RLU) were measured using a Varioskan Lux microplate reader (Thermo Scientific). Results were expressed as fold change in activity relative to the D614G pseudovirus.

### ACE2 affinity assay

The binding affinity of spike variants to biotinylated recombinant human ACE2 (biotin-rhACE2, R&D Systems; BT933) was measured as the percentage of rhACE2 bound spike expressing 293T cells. Briefly, 293T cells were transfected with individual RBD mutant spike using PEI. After 24 h of transfection, the cells were harvested and treated with 20 ng/ml of biotinylated rhACE2. Subsequently, the cells were treated with APC-conjugated streptavidin to label biotinylated rhACE2 bound cells which express RBD mutants spike proteins. Finally, APC-positive cells were analyzed by CytoFLEX flow cytometer (Beckman Coulter). To rule out the possibility that differences in spike expression level affect the affinity assay result, we performed immunoblotting using the same batch of transfected cell lysates prepared for the FACS analysis. Total spike protein levels were then quantified by summing the band intensities of the full-length (S0) and cleaved (S2) forms.

### Spike cleavage assay

Plasmids encoding RBD mutation spike of Omicron subvariants were transfected into 293T cells using PEI. After 24 h, the cells were harvested, lysed, and analyzed by immunoblotting using antibodies targeting the S2 domain of SARS-CoV-2 spike. The band intensities of full-length spike and spike S2 domain were quantified by ImageJ software (NIH) and individually normalized to the corresponding β-actin band intensity. The spike cleavage ratio was then calculated as follows: [S2 / (S2 + full-length)] × 100.

### Membrane fusion assay

The membrane fusion assay was conducted following the procedure described previously (Wang et al., 2021). VERO cells were transfected with plasmids encoding RBD mutation spike of Omicron subvariants by FuGENE HD according to the manufacturer’s instruction. After 24 h, 1 μg/ml of TPCK-trypsin was treated to each cell for 6 h followed replacing medium with fresh DMEM. After 18 h of incubation, cells were fixed with 4% paraformaldehyde and stained with Giemsa staining solution. Samples were visualized using EVOS-M5000 Imaging System (Invitrogen) and fused cells containing three or more nuclei were counted as syncytia.

### Statistical analysis

All statistical analyses were conducted with GraphPad Prism 8 software. Statistical significances were validated by Student’s t-test or one-way ANOVA and *p* < 0.05 denoted as statistical significance. Data are presented as the Mean ± SD.

## Results

### Enhanced infectivity of Omicron subvariant with RBD mutants is independent of ACE2 binding affinity

**Fig. 1.**
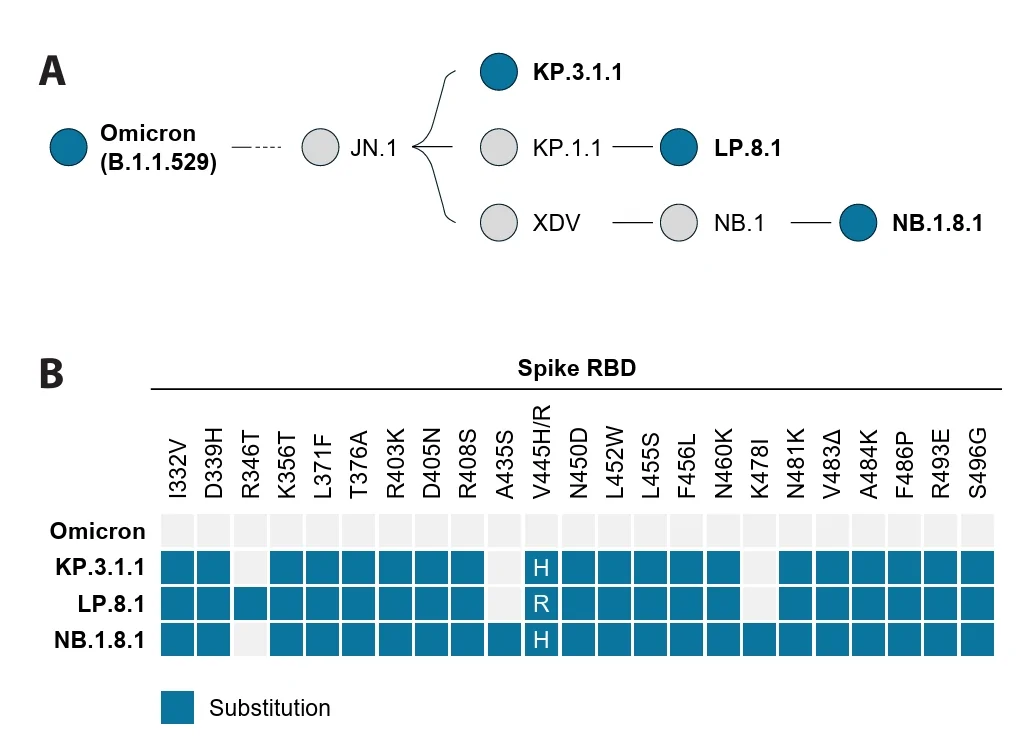
Phylogenetic tree and corresponding spike RBD substitutions of emerging Omicron subvariants. (A) A schematic diagram illustrating the evolutionary relationship of the Omicron sublineages were presented, and variants used in this study were marked with blue. (B) The key amino acid substitutions in the spike RBD of Omicron subvariant KP.3.1.1, LP.8.1, and NB.1.8.1 were presented as a blue box.

Following the global prevalence of the Omicron, several descendant sublineages classified as Variants of Interest (VOI) or Variants Under Monitoring (VUM) have emerged that likely increase the transmissibility and encode several mutations of RBD in the spike protein. To understand the functional significance of these RBD mutations, we first retrieved spike sequences for emerging Omicron subvariants: KP.3.1.1, LP.8.1, and NB.1.8.1 (Fig. 1A). Analysis of the RBD substitutions revealed a set of mutations shared among these new variants, as well as unique changes defining each lineage. For example, R346T and V445R were unique to LP.8.1, while A435S and K478I were observed only in NB.1.8.1. The defining RBD substitutions that distinguish these lineages are summarized in Fig. 1B.

**Fig. 2.**
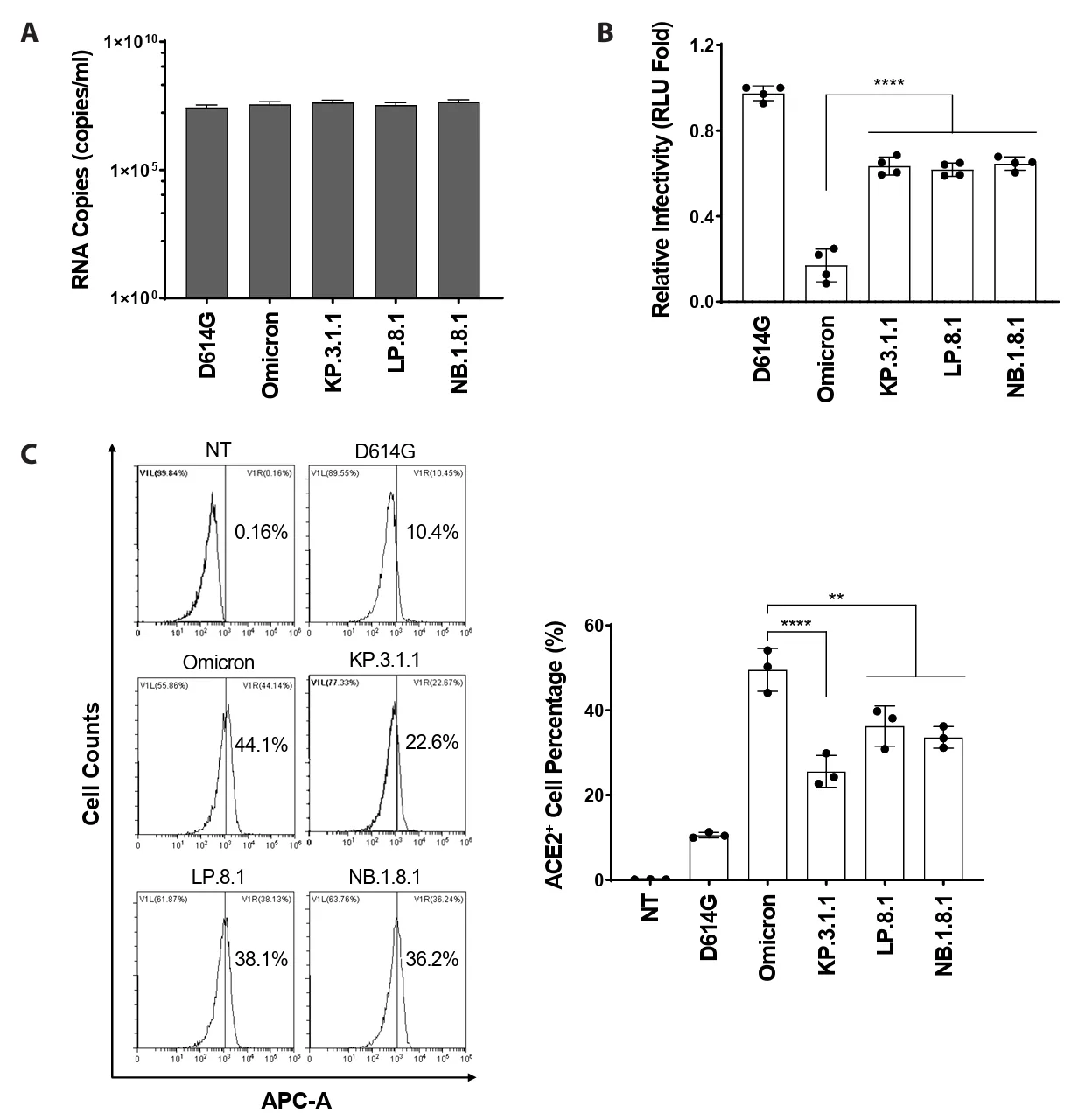
RBD mutations of emerging Omicron subvariants elicit enhanced infectivity despite reduced ACE2 binding affinity. (A) Pseudoviral RNA copies were measured by RT-qPCR with a primer pair targeting MLV 3’-LTR. The copy number of each variant was calculated with standard curve of serially diluted pQCXIP-fLuc plasmid. (B) The equal amount of each pseudovirus was transduced into 293T-ACE2-TMPRSS2 cells. Twenty-four h after transduction, relative luminescence units (RLUs) were measured. (C) 293T cells were transfected with indicated spike RBD mutants. After 24 h, biotinylated ACE2 was treated followed by Streptavidin-APC treatment. Fluorescence-bound population was quantified by flow cytometry and normalized by expression level of spike using the same batch of transfected cells. **p < 0.01, ****p < 0.0001. NT (non-transfected spike RBD mutants).

To determine how these substitutions affect entry, we generated spike constructs harboring the RBD substitutions of KP.3.1.1, LP.8.1, and NB.1.8.1 by site-directed mutagenesis and produced murine leukemia virus (MLV)-based pseudoviruses bearing each spike. We quantified MLV pseudovirus genome copies by RT-qPCR to normalize inputs across conditions (Fig. 2A), and the same amount of each pseudovirus was used for infectivity assays. As a result, pseudoviruses corresponding to KP.3.1.1, LP.8.1, and NB.1.8.1 more efficiently infected ACE2-expressing cells than the ancestral Omicron (Fig. 2B). Given that RBD mutation of spike has majorly been linked to increased receptor binding affinity, we next examined the ACE2 binding affinity of each variant spike (Harvey et al., 2021). SARS-CoV-2 spike variants containing RBD mutations were displayed on 293T cells and incubated with biotinylated ACE2 protein. Then ACE2 bound to spike on the cell surface were stained with streptavidin-APC. APC-positive cells which indicate the ACE2-Spike binding were quantified using FACS. Surprisingly, the results indicated that the KP.3.1.1, LP.8.1, and NB.1.8.1 spikes showed lower affinity than the ancestral Omicron spike (Fig. 2C). Omicron showed around 45% ACE2-positive cells, whereas KP.3.1.1, LP.8.1, and NB.1.8.1 showed only 22–35%. This suggests that the enhanced infectivity of the sublineages (Fig. 2B) is not driven by an increased ACE2 binding affinity, but rather by other entry mechanisms.

### Enhanced S1/S2 cleavage and fusogenicity compensate for reduced ACE2 affinity of Omicron subvariants

**Fig. 3.**
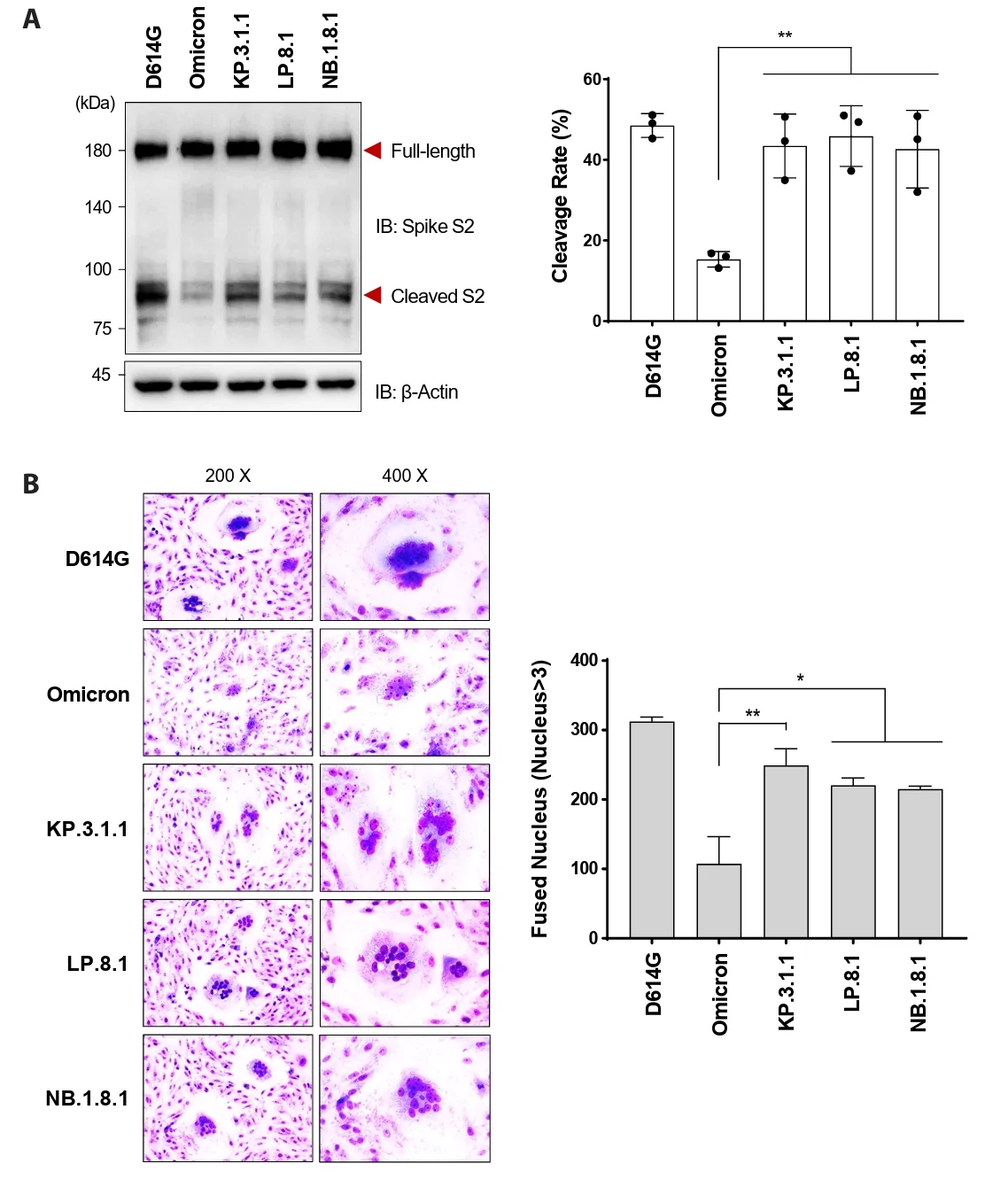
Enhanced spike cleavage efficiency and fusogenicity compensate reduced ACE2 affinity of Omicron subvariants. (A) 293T cells were transfected with plasmids expressing the indicated spike RBD mutants. At 24 h post-transfection, cell lysates were subjected to immunoblotting using an antibody targeting the spike S2 domain. The graph represents the cleavage rate. To calculate the cleavage ratio, the band intensities of the full-length spike and cleaved S2 fragment were each first normalized to the β-actin band intensity. The ratio of spike cleavage was then calculated using the following formula: [S2 / (S2 + full-length)] × 100. The β-actin blot was presented as an internal loading control. (B) Vero cells were transfected with the indicated spike variants and treated with trypsin to induce fusion. After 18 h, cells were fixed, stained, and visualized. The bar graph quantifies the number of fused nuclei from syncytia containing more than three nuclei. *p < 0.05, **p < 0.01.

An additional explanation for the higher infectivity of KP.3.1.1, LP.8.1, and NB.1.8.1 is increased S1/S2 proteolysis driven by RBD mutation. After receptor engagement, spike is primed by furin cleavage at S1/S2 and subsequent S2’ to expose the fusion peptide. Although the RBD primarily mediates receptor binding, substitutions in this region can allosterically alter spike conformation and the accessibility of the S1/S2 site (Cui et al., 2022; Park et al., 2022). We therefore quantified spike cleavage processing by expressing each spike variant in 293T cells and carried out immunoblotting with an S2-specific recognized antibody. The ratio of cleaved S2 to full-length spike was significantly higher for KP.3.1.1, LP.8.1, and NB.1.8.1 than for Omicron (Fig. 3A). To determine whether this enhanced cleavage efficiency translated to increased fusion activity, we performed a membrane fusion assay after transfection with the same spike variants. At 24 h post-transfection, the cells were treated with trypsin, followed by Giemsa staining. Consistent with the cleavage assay, cells expressing the KP.3.1.1, LP.8.1, and NB.1.8.1 spikes significantly facilitated cell-to-cell fusion as compared to cells expressing the Omicron spike protein (Fig. 3B). Taken together, these results indicate that KP.3.1.1, LP.8.1, and NB.1.8.1 promote efficient S1/S2 cleavage and subsequent cell-to-cell fusion than the ancestral Omicron. This enhanced fusogenicity provides a mechanistic explanation for the increased infectivity of KP.3.1.1, LP.8.1, and NB.1.8.1 despite possessing lower ACE2 binding affinity than Omicron.

## Discussion

The continual emergence of SARS-CoV-2 variants requires functional characterization of novel spike mutations to understand their effects on viral fitness. The RBD of SARS-CoV-2 spike is a central determinant of viral entry, yet the combined effects of substitutions in emerging Omicron subvariants remain unclear. In this study, we identified a key functional characteristic of RBD mutations in recent subvariants KP.3.1.1, LP.8.1, and NB.1.8.1. These variants display markedly higher infectivity than the ancestral Omicron (B.1.1.529) (Fig. 2B) but unexpectedly accompanied by significantly lower ACE2 binding affinity (Fig. 2C). This finding indicates that the augmented infectivity of these variants is not driven by the canonical function of RBD mutation, but rather by compensatory enhancements in post-binding steps.

After binding to ACE2, spike protein undergoes extensive conformational changes to initiate membrane fusion. Proteolytic processing of the spike protein at the S1/S2 boundary is a critical prerequisite for priming spike for the subsequent exposure of the fusion peptide (Hoffmann et al., 2020). To investigate whether the augmented infectivity of the Omicron subvariants was driven by increased spike cleavage, we next evaluated the S1/S2 cleavage efficiency of the KP.3.1.1, LP.8.1, and NB.1.8.1 spike proteins. As shown in Fig. 3A, the spike proteins of these subvariants exhibited enhanced cleavage efficiency compared to the ancestral Omicron. This observation is consistent with previous studies reporting that RBD mutations can regulate spike cleavage efficiency despite the physical distance between RBD (a.a. 319–526) and S1/S2 cleavage site (a.a. 681–685). Previously, our group showed SARS-CoV-2 Beta (B.1.351) variant-defining RBD mutations, K417N and E484K, promote efficient S1/S2 cleavage and fusogenicity (Kim et al., 2021). Conversely, the Omicron BA.1 mutations E484A, S375F, and T376A showed the opposite effect by stabilizing the spike trimer and reducing TMPRSS2 usage, thereby attenuating spike cleavage efficiency (Hu et al., 2022; Kimura et al., 2022). These contrasting examples demonstrate that RBD mutations can drive distinct conformational changes that directly influence proteolytic processing of the spike. This enhanced priming, in turn, facilitates the subsequent membrane fusion process in Omicron subvariants (Fig. 3B), thereby contributing to their increased infectivity.

Although the identified substitutions are localized within the RBD, our findings suggest that they exert allosteric control over S2 maturation and function. Structural studies have shown that RBD mutations can shift the thermodynamic equilibrium of the spike trimer; in particular, alterations that favor the 'open' RBD conformation may increase accessibility of the S1/S2 furin cleavage site to host proteases, thereby facilitating S2 maturation. Our hypothesis of allosteric regulation is further supported by the structural relevance of the mutated residues presents in these subvariants. Specifically, residue Q493 (mutated to E in KP.3.1.1) is located at a critical interface where substitutions have been shown to stabilize the RBD 'up' (open) conformation, a prerequisite for efficient ACE2 binding and subsequent proteolytic processing (Mannar et al., 2022). Similarly, residue R346 (mutated to T in LP.8.1) modulates the dynamic flexibility of the RBD core and destabilizes the 'down' state, a mechanism previously characterized in BA.4.6 and XBB lineages to facilitate immune escape (Cao et al., 2023). In the case of NB.1.8.1, the K478I mutation resides within the flexible receptor-binding ridge, and alterations at this position (e.g., T478K in Delta) have been associated with changes in loop stability and interface dynamics (Planas et al., 2021). Collectively, these mutations likely shift the thermodynamic equilibrium of the spike trimer toward an open conformation, thereby increasing exposure of the S1/S2 loop and promoting cleavage.

While our findings support the role for RBD-mediated allosteric regulation in enhancing S1/S2 cleavage, the precise substitutions driving this phenotype remain to be fully defined. Future studies employing individual mutagenesis or systematic alanine-scanning approaches will be necessary to pinpoint the causative substitutions, and structural analyses using cryo-electron microscopy (cryo-EM) will be required to directly visualize the conformational changes induced by these RBD mutations. Ideally, these mechanistic insights should be further evaluated using authentic virus models, where biosafety permits, to account for potential variations in viral entry pathways. From a therapeutic perspective, hyper-fusogenicity may pose a challenge to the efficacy of entry-targeting therapeutics. Previous work in HIV-1 has shown that viral isolates with accelerated fusion kinetics exhibit reduced sensitivity to the fusion inhibitor T-20 (Enfuvirtide), as rapid conformational transitions shorten the temporal window available for inhibitor binding (Eckert and Kim, 2001; Reeves et al., 2002). In the context of SARS-CoV-2, enhanced S1/S2 cleavage may similarly accelerate the fusion cascade, potentially reducing the potency of therapeutics that targeting transient fusion intermediates. Thus, variants with enhanced fusion capacity may necessitate a re-evaluation of therapeutic strategies and should be considered in future variant surveillance efforts.

Taking together, this study delineates a key compensatory strategy underlying the emergence of these Omicron subvariants. These lineages appear to counteract reduced receptor binding affinity through enhanced S1/S2 processing and increased membrane fusion. Our findings further underscore a non-canonical role of RBD mutation in regulating spike cleavage and fusogenicity.

## Figures and Tables

**Table 1.** Primer pairs used in this study. F; Forward, R; Reverse.

Group	Primer	Sequence
Omicron	G339D F	5'-CTGTGTCCATTTGATGAGGTGTTCAATG-3'
	G339D R	5'-CATTGAACACCTCATCAAATGGACACAG-3'
	K417N F	5'-GACAAACAGGCAACATTGCTGACTACA-3'
	K417N R	5'-TGTAGTCAGCAATGTTGCCTGTTTGTC-3'
	S371L F	5'-TCTGTGCTCTACAACCTTGCCTCCTTCAGCAC-3'
	S371L R	5'-GTGCTGAAGGAGGCAAGGTTGTAGAGCACAGA-3'
	S373P/S375F F	5'-GCTCTACAACCTTGCCCCCTTCTTCACCTTCAAGTGTTAT-3'
	S373P/S375F R	5'-ATAACACTTGAAGGTGAAGAAGGGGGCAAGGTTGTAGAGC-3'
	N440K F	5'-GGAACAGCAACAAACTGGACAGCAAGG-3'
	N440K R	5'-CCTTGCTGTCCAGTTTGTTGCTGTTCC-3'
	G446S F	5'-GGACAGCAAGGTGAGCGGCAACTACAACT-3'
	G446S R	5'-AGTTGTAGTTGCCGCTCACCTTGCTGTCC-3'
	S477N/T478K F	5'-TACCAGGCTGGCAACAAACCATGTAATGGA-3'
	S477N/T478K R	5'-TCCATTACATGGTTTGTTGCCAGCCTGGTA-3'
	E484A F	5'-TGTAATGGAGTGGCGGGCTTCAACTGT-3'
	E484A R	5'-ACAGTTGAAGCCCGCCACTCCATTACA-3'
	Q493R/G496S/Q498R F	5'-TTACTTTCCACTCAAATCCTATAGCTTCCGACCAACCAATGGA-3'
	Q493R/G496S/Q498R R	5'-TCCATTGGTTGGTCGGAAGCTATAGGATTTGAGTGGAAAGTAA-3'
	N501Y/Y505H F	5'-CTTCCAACCAACCTATGGAGTGGGCCACCAACCATACAG-3'
	N501Y/Y505H R	5'-CTGTATGGTTGGTGGCCCACTCCATAGGTTGGTTGGAAG-3'
KP.3.1.1, LP.8.1, NB.1.8.1	I332V F	5′-GAGGTTTCCAAACGTCACCAACCTGTG-3′
	I332V R	5'-CACAGGTTGGTGACGTTTGGAAACCTC-3'
	D339H F	5'-CCTGTGTCCATTTCATGAGGTGTTCAA-3'
	D339H R	5'-TTGAACACCTCATGAAATGGACACAGG-3'
	K356T F	5'-ATGCCTGGAACAGGACGAGGATTAGCAACTG-3'
	K356T R	5'-CAGTTGCTAATCCTCGTCCTGTTCCAGGCAT-3'
	L371F F	5'-TGTGCTCTACAACTTTGCCCCCTTCTT-3'
	L371F R	5'-AAGAAGGGGGCAAAGTTGTAGAGCACA-3'
	T376A F	5'-TGCCCCCTTCTTCGCCTTCAAGTGTTA-3'
	T376A R	5'-TAACACTTGAAGGCGAAGAAGGGGGCA-3'
	R403K-D405N F	5'-TCCTTTGTGATTAAGGGAAATGAGGTGAGACA-3'
	R403K-D405N R	5'-TGTCTCACCTCATTTCCCTTAATCACAAAGGA-3'
	R408S F	5'-GAAATGAGGTGAGTCAGATTGCCCCTG-3'
	R408S R	5'-CAGGGGCAATCTGACTCACCTCATTTC-3'
	N450D-L452W F	5'-CAGCGGCAACTACGACTACTGGTACAGACTGTTCA-3'
	N450D-L452W R	5'-TGAACAGTCTGTACCAGTAGTCGTAGTTGCCGCTG-3'
	L455S-F456L F	5'-CTACTGGTACAGATCGTTGAGGAAGAGCAACC-3'
	L455S-F456L R	5'-GGTTGCTCTTCCTCAACGATCTGTACCAGTAG-3'
	N460K F	5'-TGAGGAAGAGCAAGCTGAAACCATTTG-3'
	N460K R	5'-CAAATGGTTTCAGCTTGCTCTTCCTCA-3'
	N481K-V483Δ F	5'-ACAAACCATGTAAGGGAAAGGCGGGCTTCAAC-3'
	N481K-V483Δ R	5'-GTTGAAGCCCGCCTTTCCCTTACATGGTTTGT-3'
	A484K-F486P F	5'-AACCATGTAAGGGAAAGGGCCCCAACTGTTACTTTC-3'
	A484K-F486P R	5'-GAAAGTAACAGTTGGGGCCCTTTCCCTTACATGGTT-3'
	R493E F	5'-TTACTTTCCACTCGAATCCTATGGCTT-3'
	R493E R	5'-AAGCCATAGGATTCGAGTGGAAAGTAA-3'
	S496G F	5'-ACTCCAATCCTATGGCTTCCGACCAAC-3'
	S496G R	5'-GTTGGTCGGAAGCCATAGGATTGGAGT-3'
KP.3.1.1, NB.1.8.1	V445H F	5'-ACTGGACAGCAAGCACAGCGGCAACTACA-3'
	V445H R	5'-TGTAGTTGCCGCTGTGCTTGCTGTCCAGT-3'
LP.8.1	R346T F	5'-TTCAATGCCACCACGTTTGCCTCTGTC-3'
	R346T R	5'-GACAGAGGCAAACGTGGTGGCATTGAA-3'
	V445R F	5'-ACTGGACAGCAAGCGCAGCGGCAACTACG-3'
	V445R R	5'-CGTAGTTGCCGCTGCGCTTGCTGTCCAGT-3'
NB.1.8.1	A435S F	5'-AGGCTGTGTGATTTCCTGGAACAGCAA-3'
	A435S R	5'-TTGCTGTTCCAGGAAATCACACAGCCT-3'
	K478I F	5'-CAGGCTGGCAACATACCATGTAAGGGA-3'
	K478I R	5'-TCCCTTACATGGTATGTTGCCAGCCTG-3'
